# Reproducibility and differences in area of foveal avascular zone measured by three different optical coherence tomographic angiography instruments

**DOI:** 10.1038/s41598-017-09255-5

**Published:** 2017-08-29

**Authors:** Hideki Shiihara, Taiji Sakamoto, Takehiro Yamashita, Naoko Kakiuchi, Hiroki Otsuka, Hiroto Terasaki, Shozo Sonoda

**Affiliations:** Department of Ophthalmology, Kagoshima University Graduate School of Medical and Dental Sciences, Kagoshima, Japan

## Abstract

This study was performed to compare the area of the foveal avascular zone (FAZ-area) obtained by three optical coherence tomography angiography (OCTA) instruments. This was a cross-sectional, non-interventional study of twenty-seven healthy right eyes. The superficial and deep FAZ-area was measured manually with three OCTA instruments: Triton (Topcon), RS3000 (Nidek), and CIRRUS (Zeiss). The intra-rater, inter-rater, and inter-instrument correlation coefficients (CC) were assessed. The intra-rater and inter-rater CC were significantly high for the superficial and deep FAZ-areas (*P* < 0.001). The inter-instrument CC (95% confidence interval) for the superficial FAZ-area was 0.920 (0.803–0.965) for Triton vs RS3000, 0.899 (0.575–0.965) for RS3000 vs CIRRUS, and was 0.963 (0.913–0.983) for CIRRUS vs Triton (*P* < 0.001). For the deep FAZ-area, the inter-instrument CC was 0.813 (0.633–0.910) for Triton vs RS3000, 0.694 (0.369–0.857) for RS3000 vs CIRRUS, and 0.679 (0.153–0.872) for CIRRUS vs Triton (*P* < 0.001). The superficial FAZ-area (mm^2^) was 0.264 ± 0.071 with Triton, 0.278 ± 0.072 with RS3000 and 0.257 ± 0.066 with CIRRUS. For deep FAZ-area, it was 0.617 ± 0.175 with Triton, 0.646 ± 0.178 with RS3000 and 0.719 ± 0.175 with CIRRUS. The FAZ-area from these instruments was clinically interchangeable. However, the absolute values of FAZ-area are significantly different among them. These differences must be considered in comparing the FAZ-areas from different OCTA instruments.

## Introduction

Fluorescein angiography (FA) and indocyanine green angiography (ICGA) have proven to be very good methods to evaluate retinal diseases^1–3^. They have provided large amounts of information to the understanding and treatments of many retinal diseases. However, there are substantial drawbacks of FA and ICGA including their invasiveness which has made their use less frequent in the clinic^4, 5^.

Optical coherence tomography angiography (OCTA) is a newly developed non-invasive imaging technique that is based on motion contrast imaging of high-resolution volumetric blood flow to generate angiographic images in a matter of seconds^6^. Its potential clinical application is large although there is very little level I evidence of its reproducibility. For example, there is a non-vascular area in the fovea known as the foveal avascular zone (FAZ) which can be deformed in diabetic retinopathy, age-related macular degeneration, and retinal vascular occlusion^7–14^. Although the FAZ has already been detected by FA, the ability of OCTA to detect the microvascular structures clearly with little invasiveness makes the size and shape of the FAZ a clinical parameter to investigate in different retinal diseases^7–14^. However, the algorithms used in the commercially-available OCTA instruments to record images are believed to be different among the instruments. Thus, the FAZ area determined by the different OCTA instruments might not be interchangeable. The issue of interchangeability among the different devices was reported on the other OCT parameters, e.g., choroidal thickness, foveal structures, and thickness^15–17^. Considering the potential usefulness of OCTA in clinical ophthalmology, it is necessary to know the characteristics of each instrument including the inter-examination and inter-instrument reproducibility. To the best of our knowledge, there has not been a study comparing the OCTA measurements obtained in a standardized and prospective way by three different commercially-available instruments.

Thus, the aim of this study was to determine the inter-instrument reproducibility of the measurements made by three commercially-available OCTA instruments on normal eyes. Because the area of the FAZ is an easily detectable structure in the OCTA images, it was analyzed. In addition, the intra-rater and inter-rater correlation coefficients of 3 independent masked graders were determined.

## Results

Forty volunteers were screened for this study but 8 eyes were excluded because of high myopia, cataract, or corneal cloudiness. Of the 32 eyes studied, 27 had clear OCTA images (19 men and 8 women). Their average age was 36.8 ± 10.2 years ( ± SD) with a range from 26 to 57 years. The average refractive error was slightly myopic at −2.7 ± 1.6 D with a range from −0.25 to −5.25 D. The average IOP was 13.9 ± 2.2 mmHg with a range from 10 to 20 mmHg.

### Repeatability of examinations: intra-class or inter-class correlation coefficients

The coefficient of variance ranged from 0.24 to 0.29 for the 3 instruments for the superficial FAZ and the deep FAZ. For each instrument, the intra-rater correlation coefficient was significantly high and almost perfectly matched the intraclass correlation coefficient (ICC) or was higher than 0.987 (*P* < 0.001, Table 1). The inter-rater correlation coefficient was also high and almost perfectly matched the ICC by being higher than 0.964 (*P* < 0.001, Table 2). This held for the superficial and the deep FAZs.

**Table 1:** Intra-rater comparison.

FAZ	Triton			RS3000			CIRRUS		
	ICC (95%CI)	CV	P value	ICC	CV	P value	ICC	CV	P value
Superficial	0.987 (0.972–0.994)	0.26	<0.001	0.987 (0.972–0.994)	0.28	<0.001	0.991 (0.981–0.996)	0.26	<0.001
Deep	0.990 (0.978–0.995)	0.29	<0.001	0.991 (0.980–0.996)	0.28	<0.001	0.995 (0.989–0.998)	0.24	<0.001

FAZ, foveal avascular zone; ICC, intraclass correlation coefficients; CV, coefficient of variation.

**Table 2:** Inter-rater comparison.

FAZ	Triton			RS3000			CIRRUS		
	ICC (95%CI)	CV	P value	ICC	CV	P value	ICC	CV	P value
Superficial	0.973 (0.941–0.987)	0.28	<0.001	0.964 (0.922–0.983)	0.28	<0.001	0.986 (0.969–0.994)	0.26	<0.001*
Deep	0.992 (0.983–0.996)	0.28	<0.001	0.981 (0.960–0.996)	0.27	<0.001	0.986 (0.969–0.994)	0.25	<0.001

FAZ, foveal avascular zone; ICC, intraclass correlation coefficients; CV, coefficient of variation.

### Inter-instrument comparisons

The area of the FAZ determined by the 3 instruments were significantly correlated with each other (Table 3). For the superficial FAZ, the intra-class correlation coefficient was greater than 0.89 for any combination of the three instruments. For the deep FAZ, the intra-class correlation coefficient was good at ≥0.67 for any combination but weaker than that for the superficial FAZ (Table 4).

**Table 3:** Comparison of two instruments for superficial FAZ.

	Mean Difference, mm^2^ ± SD	Range of Difference, mm^2^	95% CI	P value Wilcoxon signed rank test	ICC	95% CI	P value
Triton -RS3000	−0.013 ± 0.026	−0.076 to 0.045	−0.024 to −0.003	0.012	0.92	0.803–0.965	<0.001
RS3000 -CIRRUS	0.021 ± 0.025	−0.030 to 0.064	0.011 to 0.031	<0.001	0.899	0.575–0.965	<0.001
CIRRUS -Triton	−0.007 ± 0.180	−0.048 to 0.026	−0.015 to 0.000	0.049	0.963	0.913–0.983	<0.001

CI, confidence interval; ICC, intraclass correlation coefficients.

**Table 4:** Comparison of two instruments for deep FAZ.

	Mean Difference mm^2^ ± SD	Range of Difference mm^2^	95% CI	P value Wilcoxon signed rank test	ICC	95% CI	P value
Triton -RS3000	−0.030 ± 0.108	−0.344 to 0.142	−0.073 to 0.013	0.230	0.813	0.633–0.910	<0.001
RS3000 -CIRRUS	−0.073 ± 0.129	−0.382 to 0.245	−0.124 to −0.022	0.006	0.694	0.369–0.857	<0.001
CIRRUS -Triton	0.103 ± 0.117	−0.216 to 0.372	0.056 to 0.149	<0.001	0.679	0.153–0.872	<0.001

CI, confidence interval; ICC, intraclass correlation coefficients.

The average area of the superficial FAZ was 0.264 ± 0.071 mm^2^ measured with the Triton, 0.278 ± 0.072 mm^2^ measured with the RS3000, and 0.257 ± 0.066 mm^2^ measured with the CIRRUS. The area of the superficial FAZ obtained with the Triton was 0.013 mm^2^ smaller than the RS3000 which was significantly different (*P* = 0.012). The superficial FAZ measured with the RS3000 was 0.021 mm^2^ larger than that with the CIRRUS which was significantly different (*P* < 0.001). The superficial FAZ measured with the CIRRUS was 0.007 mm^2^ smaller than that of the Triton (*P* = 0.049). The maximum values for the differences were −0.076 mm^2^ for the Triton minus the RS3000, 0.064 mm^2^ for the RS3000 minus the CIRRUS, and −0.048 mm^2^ for the CIRRUS minus the Triton (Table 3).

The average area of the deep FAZ was 0.617 ± 0.175 mm^2^ measured with the Triton, 0.646 ± 0.178 mm^2^ measured with the RS3000, and 0.719 ± 0.175 mm^2^ measured with the CIRRUS. The area of the deep FAZ obtained with the Triton was 0.030 mm^2^ smaller than that with the RS3000, but the difference was not significant (*P* = 0.23). The deep FAZ measured with the RS3000 was 0.073 mm^2^ smaller than that with the CIRRUS which was significant (*P* < 0.001). The deep FAZ measured with the CIRRUS was 0.103 mm^2^ larger than that of the Triton which was significant (*P* = 0.049).The maximum values for the differences were −0.334 mm^2^ for the Triton minus the RS3000, −0.382 mm^2^ for the RS3000 minus the CIRRUS, and 0.372 mm^2^ for the CIRRUS minus the Triton (Table 4).

In CIRRUS, the area of the superficial FAZ determined by the embedded automated software was 0.253 ± 0.068 mm^2^ which was highly comparable to the area measured manually at 0.257 ± 0.066 mm^2^ (*P* < 0.001; ICC: 0.987 Confidence interval; 0.970–0.994).

### Bland-Altman plot analyses

**Figure 1:**
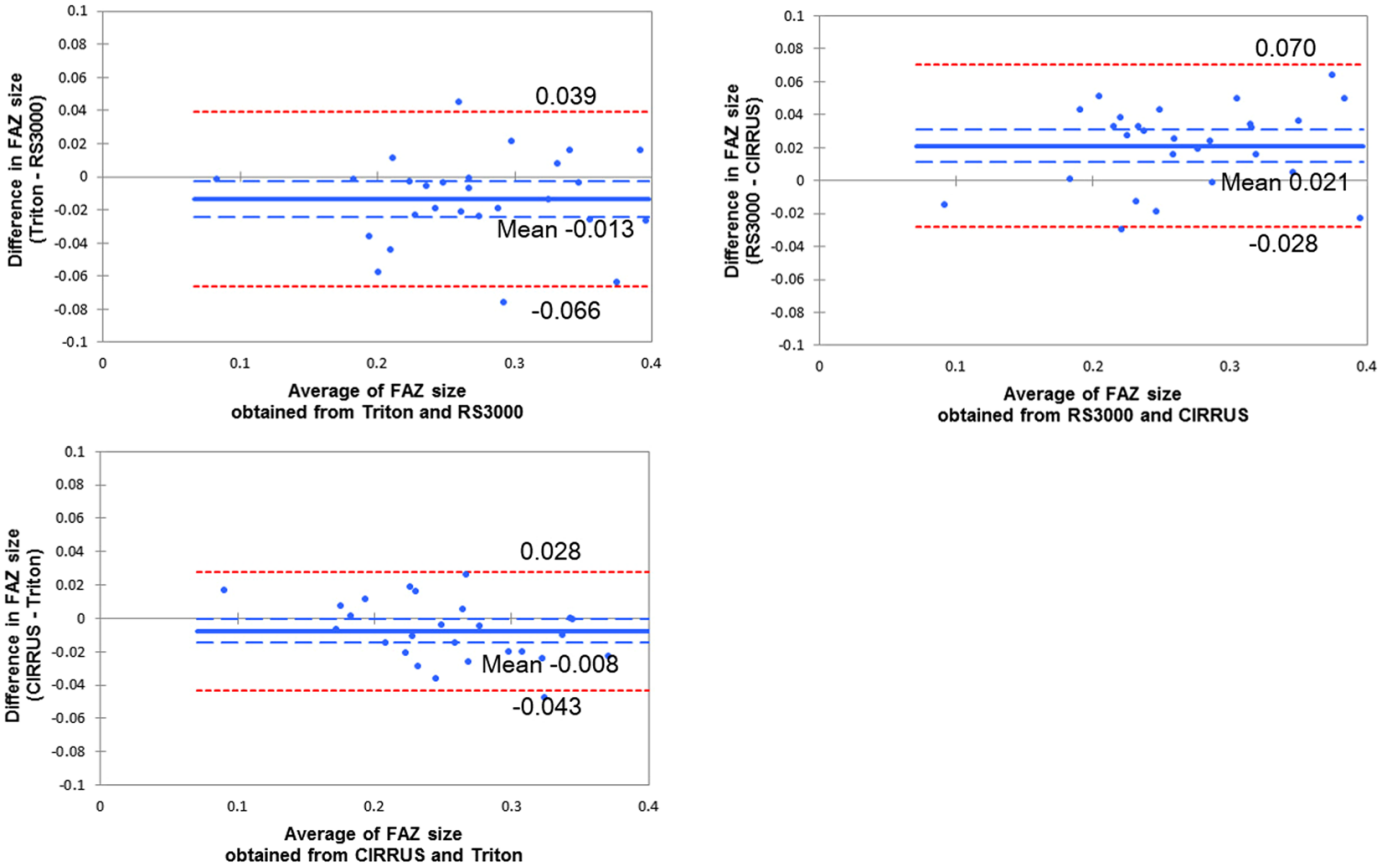
Bland-Altman plot analyses for the superficial FAZ. There was a fixed bias among the three instruments but there was no proportional bias between any two instruments.

For the superficial FAZ, there was a fixed bias between the instruments; the Triton vs the RS3000 had a bias (95% CI, −0.024 to −0.003), and for the RS3000 vs the CIRRUS had a bias (95% CI, 0.011 to 0.031), and the CIRRUS vs the Triton had a bias (95% CI, −0.015 to −0.00). While there was not any proportional bias between any two instruments, the Triton vs the RS3000 had a regression coefficient of 0.020 (*P* = 0.991), the RS3000 vs the CIRRUS had a regression coefficient of 0.22 (*P* = 0.27), and the CIRRUS vs the Triton had a regression coefficient 0.306 (*P* = 0.12; Fig. 1).

**Figure 2:**
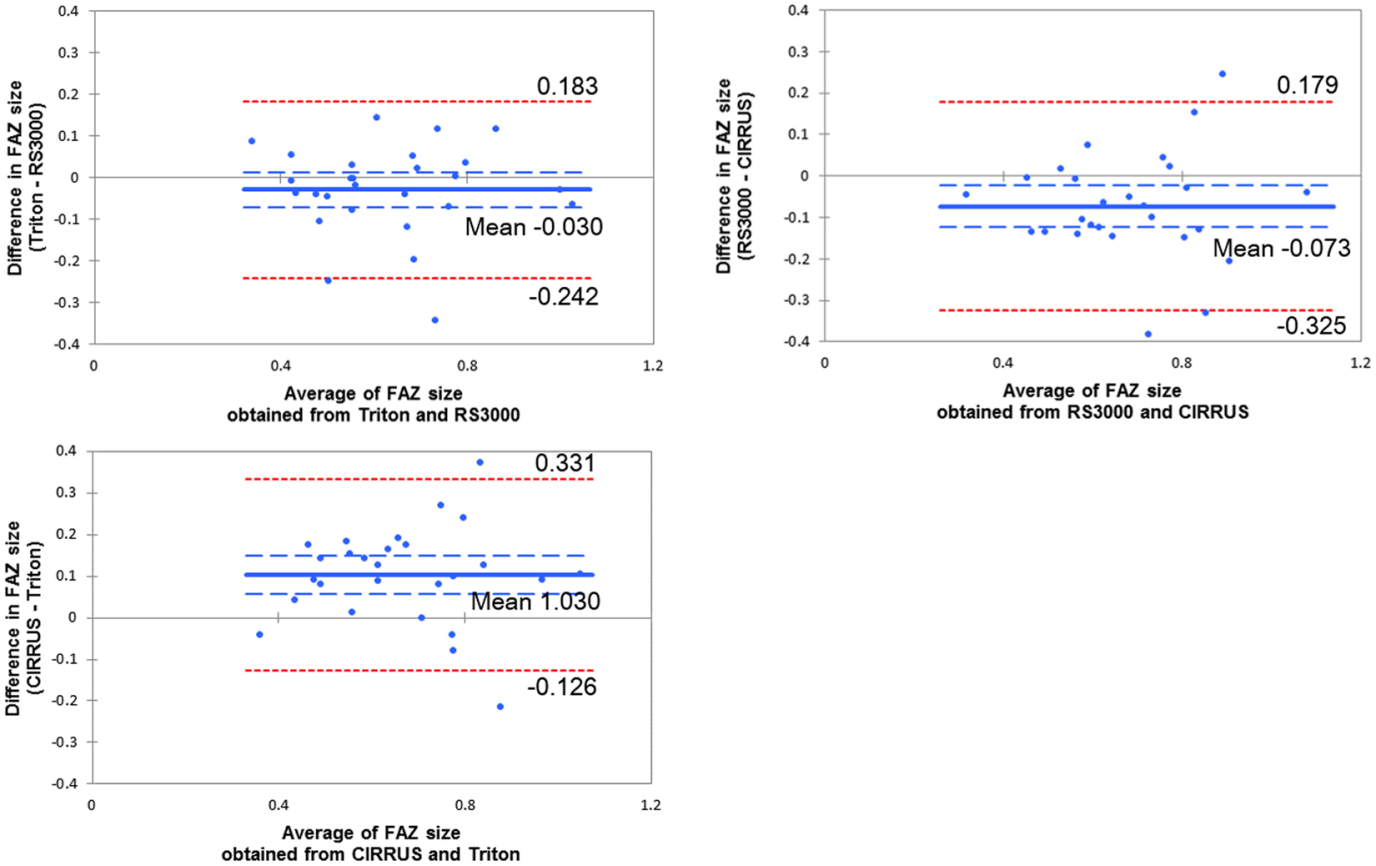
Bland-Altman plot analysis for the deep FAZ. There was a fixed bias between the RS3000 and CIRRUS and between the CIRRUS and Triton. However, there was no fixed bias between the Triton and RS3000. There was no proportional bias between any two machines.

For the deep FAZ, there was a fixed bias between the RS3000 and the CIRRUS (95% CI, −0.124 to −0.022), or the CIRRUS and the Triton (95% CI, 0.056 to 0.149). But there was no fixed bias between the Triton and the RS3000 (95% CI, −0.073 to 0.013). There was no proportional bias between any two instruments; the Triton vs the RS3000, (regression coefficient 0.026; *P* = 0.898), the RS3000 vs the CIRRUS (regression coefficient 0.022; *P* = 0.913) and the CIRRUS vs the Triton (regression coefficient 0.001; *P* = 0.993; Fig. 2).

### Comparisons of 3 × 3 images of superficial FAZ of the same eye

**Figure 3:**
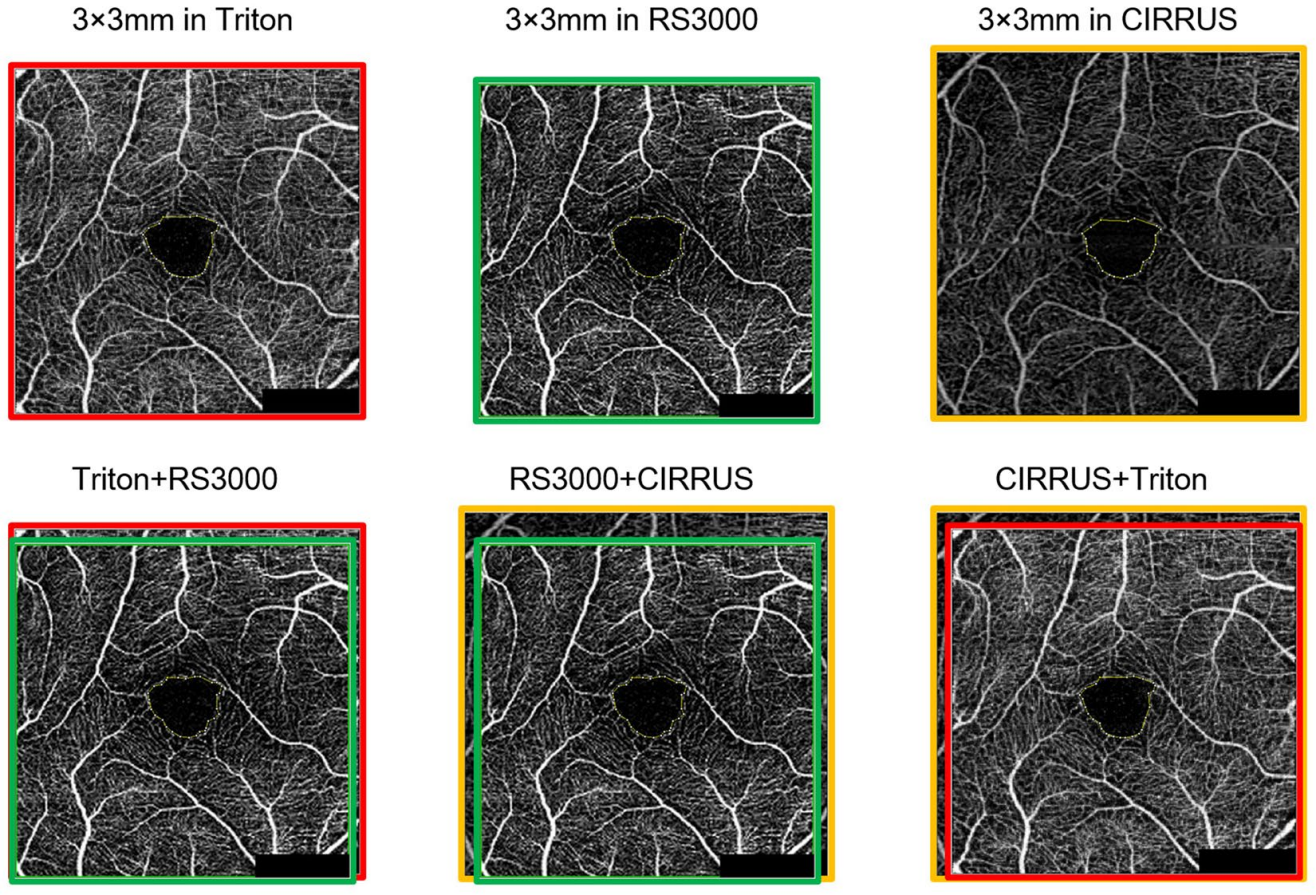
Comparison of 3 × 3 images of superficial foveal avascular zone (FAZ) of the same eye obtained by the Triton, RS3000, and CIRRUS optical coherence tomography angiographic (OCTA) instruments. Representative 3 × 3 retina OCTA images obtained from the same eye with the Triton instrument (top left with red bordering), RS3000 (top middle with green bordering) and CIRRUS (top right with yellow bordering). The superimposed FAZ images are shown in the bottom row. Left; Triton and RS3000, middle; RS3000 and CIRRUS, right; CIRRUS and Triton. The CIRRUS FAZ image is always the largest area among the three instruments.

The area of the FAZ measured by the CIRRUS was always the largest among the three instruments (Fig. 3). The Triton defined the area larger than the RS3000, except for one eye. In the case that the RS3000 was larger, a slight motion artifact was recognized for the Triton. Therefore, even though the reference scale was set at 3 mm on the retina, its actual size was not the same for the three instruments. The percentage of images measured with the RS3000 was 90.0 ± 0.02% and that with the Triton was 96.6 ± 0.01% of that measured with the CIRRUS.

## Discussion

The results showed that the intra-rater and inter-rater correlation coefficients were high for any single instrument indicating that each is clinically suitable for evaluating the area of the FAZs. The inter-instrument correlation coefficients were also high for the superficial FAZ, however the absolute value of the FAZ was significantly different between instruments with a significant fixed bias. For example, the value of the superficial FAZ measured with the RS3000 was 0.013 mm^2^ larger than that of the Triton, the RS3000 was 0.021 mm^2^ larger than that by the CIRRUS and the Triton was 0.007 mm^2^ larger than that of the CIRRUS. For the deep FAZ, the inter-instrument correlation coefficient was also good but it was lower than that for the superficial FAZ. The absolute value of the area of the deep FAZ was significantly different between the CIRRUS and the RS3000 or between the CIRRUS and the Triton but not between the Triton and the RS3000.

We investigated the reason why these differences were present. By merging the 3 × 3 mm images of the superficial slab of the same eye obtained with different instruments, it was found that the CIRRUS defined the 3 × 3 mm area as being larger than that of the other two instruments (Fig. 3). As a result, the area of the FAZ was calculated to be the smallest with the CIRRUS among the three instruments. This was further confirmed by Brand-Altman analysis that showed the presence of fixed bias between instruments. In the 3 × 3 images defined by each instrument, the Triton was on the average 96.6% of the CIRRUS, and the RS3000 was 90.0% of the CIRRUS. Because the average superficial FAZ was 0.278 mm^2^ with the RS3000, it can be calculated to be 0.250 mm^2^ with the CIRRUS (0.278 × 90/100), which was almost the same as the actual FAZ of 0.252 mm^2^ with the CIRRUS. The average superficial FAZ was 0.264 mm^2^ with the Triton, so it should be 0.255 mm^2^ theoretically with the CIRRUS (0.264 × 96.6/100), which was also the same as the actual FAZ with the CIRRUS. This difference of the 3 × 3 mm area size between instruments can clearly explain the difference of FAZ sizes. It is possible that these differences were made by the magnification effect caused by the differences in the axial length. Thus, the larger area was present in eyes with longer axial lengths without adjustments. Because the CIRRUS does not make adjustments for the axial length, it may be possible due to the magnification effect.

An important finding was that the inter-instrument correlation coefficient was very high for the superficial FAZ but not for the deep FAZ. In previous studies using a single instrument, the repeatability was almost perfect for the superficial FAZ but not for the deep FAZ^18–21^. Indeed, the average superficial FAZ of normal subjects was ranged from 0.250 to 0.304 mm^2^ with 0.054 mm^2^ difference, while the size of the deep FAZ was ranged from 0.34 to 0.614 mm^2^ with 0.274 mm^2^ difference, in which the difference was larger than that for the superficial FAZ^14, 18–21^. The cause for the wide differences on the quantitative information of the deep plexus can probably be explained by the influence of shadow artifacts^20^. Qualitatively, the FAZ borders were better defined at the level of the superficial than the deep network. This is anatomically reasonable because these capillary networks branch off from the parafoveal capillaries in the ganglion cell layer, and they form the superficial FAZ in agreement with early histological studies^22, 23^. In addition, the size of the superficial FAZ has a very strong correlation with the FAZ in the full-thickness retina although deep FAZ does not^24^. This suggests that the superficial FAZ can be detected more clearly than the deep FAZ. This also supports our results.

Another explanation for the indistinct borders of the deep FAZ may be the spatial resolution of the imaging instruments. The currently available OCTA instruments can identify the superficial and deep retinal vascular networks. However, histological studies show that the deep network is composed of 2 individual and separate networks which could affect the definition of FAZ border by each device^24, 25^. This is supported by the present findings of the differences in the fixed biases between the superficial and deep FAZ. In the superficial FAZ, the FAZ was largest with the RS3000 followed by the Triton and the CIRRUS. Even though the measured C-scan area was exactly the same with the same instruments, the deep FAZ measured with the CIRRUS was larger than that by the Triton which was the opposite for the superficial FAZ. Even though the defined 3 × 3 mm image area with the CIRRUS would be larger than with the other instruments, the relatively indistinct borders of the deep FAZ might make it more unclear. Because the algorithm equipped with each instrument is not fully open sourced, it is difficult to determine the exact reason for these differences.

There are several limitations in this study. First, the number of subjects was not large. Second, the subjects were healthy but myopic. Because young Japanese individuals are one of the most myopic populations the present results probably do not represent the real world data^26^. Third, the analysis was of a small field of view of 3 × 3 mm^2^. A larger field of view using OCTA may allow further information such as the correlation between FAZ area and changes in the periphery.

Another major limitation of the OCTA technology is the accuracy of the segmentation algorithm equipped with each instrument. Although these studies were on healthy subjects, segmentation errors were present in some of the eyes. The automatic segmentation or the development of a more efficient automatic correction algorithm would be preferable. In addition, the automatic software used to measure the FAZ area is not available for RS3000 and Triton OCTA instruments. Thus, we analyzed FAZ area manually with ImageJ. Indeed, most of published papers investigating the FAZ area in the OCTA images use the ImageJ software as we did in this study^7, 20, 24, 27–29^. The development of an automated software might make this analysis easier and more accurate. Under these conditions, it is important for researchers and clinicians to know the 3 × 3 mm area defined by each machine is not always the same. This information must be shared in the ophthalmological society.

In conclusion, considering that the Bland-Altman plots were distributed within the limits of agreement, the area of the superficial FAZ determined by these instruments can be clinically interchangeable to a certain extent. However, the absolute values of FAZ areas are significantly different from each other. This should be remembered when interpreting the results measured with different instruments, such as the longitudinal size or among the different cohorts, and when the data collected in different clinics using different instruments are compared. Additionally, the information obtained in this study should be shared between different manufacturers for further improvements.

## Methods

All of the procedures used conformed to the tenets of the Declaration of Helsinki. A signed informed consent was obtained from all of the subjects after an explanation of the procedures to be used. This was a prospective, cross sectional study that was performed at a single institution from April 30 to May 30, 2016. The procedures used in this study were approved by the Ethics Committee of Kagoshima University Hospital (Kagoshima, Japan) and registered with the University Hospital Medical Network (UMIN)-clinical trials registry (CTR). The registration title was, “UMIN000024919, Analysis of retinal blood vessels and blood flow using optical coherence tomography”. A detailed protocol is available at https://upload.umin.ac.jp/cgi-open-bin/ctr/ctr_view.cgi?recptno = R000028514.

Approximately 40 volunteers were initially enrolled, and all had a complete ocular examination including measurements of the refractive error (spherical equivalent) with an autorefractor keratometer (RM8900; Topcon, Tokyo, Japan), best-corrected visual acuity (BCVA), IOP with a pneumotonometer (CT-80; Topcon), slit-lamp biomicroscopy, and dilated funduscopy. The axial length was measured by optical interferometry (OA-1000; Tomey, Tokyo, Japan). Only the right eye was measured with the three OCT angiographic instruments.

The inclusion criteria were an age of >20 years and <60 years, BCVA equal or better than 20/20, and normal fundus by ophthalmoscopy and OCT. The exclusion criteria were history of ocular and systemic diseases, prior ocular surgery or intraocular injections, and high myopia of −6.0 diopters (D) or higher.

### Optical Coherence Tomography Angiography (OCTA) Scanning Protocol

All OCTA examinations were performed based on the analysis protocol and variables for each device (Table 5). Following each examination, the best image was projected onto a computer screen and evaluated by 3 independent masked graders (HS, NK, SS). If the OCTA image was determined to be clear and the FAZ distinguishable by at least 2 of the graders, the image was used for the following examinations.

**Table 5:** Scanning Protocols of OCTA instruments.

	Optical source wave length (nm)	Scan speed (A-Scan/sec)	Axial resolution (μm)	Transversal resolution (μm)	Axial adjustment
Triton	1,050	100,000	8	20	available
RS3000	880	53,000	7	20	available
CIRRUS	840	68,000	5	15	unavailable

For the intra-rater agreement analysis, two OCTA images were collected by HS within a 30 minute period. For the inter-rater agreement analysis, the OCTA images were taken of each of the patients by two examiners (HS, NK) and evaluated.

#### Measurements with the Triton instrument

SS OCT Angio OCTA software of the commercially available DRI-OCT Triton (Topcon) was operated as reported in detail^8, 27^. The images were obtained using a swept-source OCT device with a central wavelength of 1050 nm, an acquisition speed of 100,000 A-scans/sec, and an axial and transversal resolution of 8 and 20 μm in tissue, respectively. Scans were taken from 3 × 3 mm cubes with each cube consisting of 320 clusters of four repeated B-scans centered on the fovea. Based on these default settings, the superficial network extended from 2.6 μm below the internal limiting membrane (ILM) to 15.6 μm below the inner plexiform layer (IPL). The deep capillary network extended from 15.6 to 70.2 μm below the IPL. The axial adjustment was done using the software equipped with the device.

#### Measurements with the RS3000 instrument

Angio Scan OCTA software of the commercially available RS 3000 Advance (Nidek, Tokyo, Japan) was operated. The images were obtained using a SD-OCT device with a central wavelength of 880 nm, an acquisition speed of 53,000 A-scans/sec, and an axial and transversal resolution of 7 and 20 μm in tissue, respectively. Scans were taken from 3 × 3 mm cubes with each cube consisting of 256 clusters of four repeated B-scans centered on the fovea. Based on these default settings, the superficial network extended from the top of the ILM to 8 μm below the IPL. The deep capillary network extended from 13 to 88 μm below the IPL. The axial adjustment was done using the software equipped with this device.

#### Measurements with the CIRRUS instrument

Angio Plex OCTA software of the commercially available CIRRUS HD-OCT models 5000 (Carl Zeiss Meditech, Dublin, California, USA)^30^. The images were obtained using a SD-OCT device with a central wavelength of 840 nm, an acquisition speed of 68,000 A-scans/sec, and an axial and transversal resolution of 5 and 15 µm in tissue, respectively. Scans were taken from 3 × 3 mm cubes with each cube consisting of 245 clusters of four repeated B-scans centered on the fovea. Based on these default settings, the superficial capillary network was generated separated using the automated software algorithm. The deep capillary network extended from the lower end of the superficial layer to 70 μm below the IPL. The axial adjustment was not done.

### Image analyses

The FAZ area was defined as the avascular area in the center of the fovea, and the border of the FAZ was manually drawn by 2 retina specialists (HS and NK) who were masked to the clinical information. These examiners started with the raw images, did their own segmentation correction, and then analyzed the images. The ImageJ software was used to calculate the area of the FAZ as reported^14^. To delineate the border of the FAZ, “Polygon selections” program was used to demarcate the boundaries of the FAZ manually.

### Comparison of manual and automated measurement of FAZ area in CIRRUS

There was no commercially available automated software embedded in the OCTA instruments to measure the FAZ area when we conducted this study. Very recently, a software became available for the CIRRUS (superficial FAZ). We thus compare the superficial FAZ area measured manually or by the software in Zeiss OCTA. The superficial FAZ area in the images used in this study was measured with the embedded software. The absolute value of the area was compared with the results by manual measurement.

### Merging FAZ images of different OCTA instruments

The OCTA images from each instrument were used. For the Triton instrument, the reference was determined by the properties of the instrument with 256 pixels representing 3 mm. For the RS3000, 345 pixels represented 3 mm, and for the CIRRUS, 429 pixels represent 3 mm. These values were converted to mm^2^ using a scale conversion. They were then merged based on the distribution of the retinal vessels.

The ranking of the size of each image was determined by 2 of the masked examiners (HS, TY; Fig. 3). Because our preliminary study showed that the CIRRUS always had the largest area, the areas measured with the RS3000 or the Triton were expressed as the percentage of the area measured with the CIRRUS for each eye. Then, the percentage was averaged for each device.

### Statistical Analyses

All statistical analyses were performed with SPSS statistics 19 for Windows (SPSS Inc., IBM, Somers, New York, USA). The coefficient of variation (SD/mean) also was calculated. The intra-rater correlation coefficients were calculated using 1-way random effects model for measurements of agreement. Inter-instrument and inter-rater correlation coefficients were calculated using 2-way mixed-effects model for measurements of absolute agreement. Because of the clinical importance, the comparisons often were performed between two instruments^15, 16^. Therefore, the FAZ measurements from each instrument were compared to those of two of the three instruments using Wilcoxon signed rank test. Bland-Altman plots were generated to assess agreement of measurements between two of the three OCTA instruments. Differences in FAZ between instruments were plotted against mean FAZ measurements on these graphs. *P* value less than 0.05 was considered to be statistically significant.

## References

[1] Do DV (2013). Detection of new-onset choroidal neovascularization. Curr Opin Ophthalmol.

[2] Kotsolis AI, Killian FA, Ladas ID, Yannuzzi LA (2010). Fluorescein angiography and optical coherence tomography concordance for choroidal neovascularisation in multifocal choroidtis. Br J Ophthalmol.

[3] Stanga, P. E., Lim, J. I. & Hamilton, P. Indocyanine green angiography in chorioretinal diseases: indications and interpretation: an evidence-based update. *Ophthalmology* **110**, 15–21; quiz 22–13 (2003). 10.1016/s0161-6420(02)01563-4 12511340

[4] Garski TR, Staller BJ, Hepner G, Banka VS, Finney RA (1978). Adverse reactions after administration of indocyanine green. JAMA.

[5] Ha SO, Kim DY, Sohn CH, Lim KS (2014). Anaphylaxis caused by intravenous fluorescein: clinical characteristics and review of literature. Intern Emerg Med.

[6] Spaide RF, Klancnik JM, Cooney MJ (2015). Retinal vascular layers imaged by fluorescein angiography and optical coherence tomography angiography. JAMA Ophthalmol.

[7] Adhi M (2016). Retinal Capillary Network and Foveal Avascular Zone in Eyes with Vein Occlusion and Fellow Eyes Analyzed With Optical Coherence Tomography Angiography. Invest Ophthalmol Vis Sci.

[8] Al-Sheikh M, Akil H, Pfau M, Sadda SR (2016). Swept-Source OCT Angiography Imaging of the Foveal Avascular Zone and Macular Capillary Network Density in Diabetic Retinopathy. Invest Ophthalmol Vis Sci.

[9] Balaratnasingam C (2016). Visual Acuity Is Correlated with the Area of the Foveal Avascular Zone in Diabetic Retinopathy and Retinal Vein Occlusion. Ophthalmology.

[10] Coscas F (2016). Optical Coherence Tomography Angiography in Retinal Vein Occlusion: Evaluation of Superficial and Deep Capillary Plexa. Am J Ophthalmol.

[11] de Carlo TE (2015). Detection of Microvascular Changes in Eyes of Patients with Diabetes but Not Clinical Diabetic Retinopathy Using Optical Coherence Tomography Angiography. Retina.

[12] Di G (2016). A morphological study of the foveal avascular zone in patients with diabetes mellitus using optical coherence tomography angiography. Graefes Arch Clin Exp Ophthalmol.

[13] Kashani AH, Lee SY, Moshfeghi A, Durbin MK, Puliafito CA (2015). Optical Coherence Tomography Angiography of Retinal Venous Occlusion. Retina.

[14] Takase N (2015). Enlargement of Foveal Avascular Zone in Diabetic Eyes Evaluated by En Face Optical Coherence Tomography Angiography. Retina.

[15] Matsuo Y (2013). Comparisons of choroidal thickness of normal eyes obtained by two different spectral-domain OCT instruments and one swept-source OCT instrument. Invest Ophthalmol Vis Sci.

[16] Pierro L, Giatsidis SM, Mantovani E, Gagliardi M (2010). Macular thickness interoperator and intraoperator reproducibility in healthy eyes using 7 optical coherence tomography instruments. Am J Ophthalmol.

[17] Terasaki H (2012). Comparison of foveal microstructure imaging with different spectral domain optical coherence tomography machines. Ophthalmology.

[18] Carpineto P (2016). Reproducibility and repeatability of foveal avascular zone measurements in healthy subjects by optical coherence tomography angiography. Br J Ophthalmol.

[19] Mammo Z (2015). Quantitative Noninvasive Angiography of the Fovea Centralis Using Speckle Variance Optical Coherence Tomography. Invest Ophthalmol Vis Sci.

[20] Shahlaee A, Pefkianaki M, Hsu J, Ho AC (2016). Measurement of Foveal Avascular Zone Dimensions and its Reliability in Healthy Eyes Using Optical Coherence Tomography Angiography. Am J Ophthalmol.

[21] Yu J (2015). Macular perfusion in healthy Chinese: an optical coherence tomography angiogram study. Invest Ophthalmol Vis Sci.

[22] Snodderly DM, Weinhaus RS, Choi JC (1992). Neural-vascular relationships in central retina of macaque monkeys (Macaca fascicularis). J Neurosci.

[23] Weinhaus RS, Burke JM, Delori FC, Snodderly DM (1995). Comparison of fluorescein angiography with microvascular anatomy of macaque retinas. Exp Eye Res.

[24] Tan CS (2016). Optical Coherence Tomography Angiography Evaluation of the Parafoveal Vasculature and Its Relationship With Ocular Factors. Invest Ophthalmol Vis Sci.

[25] Tan PE (2015). Quantitative Comparison of Retinal Capillary Images Derived By Speckle Variance Optical Coherence Tomography With Histology. Invest Ophthalmol Vis Sci.

[26] Sawada A (2008). Refractive errors in an elderly Japanese population: the Tajimi study. Ophthalmology.

[27] Iafe NA, Phasukkijwatana N, Chen X, Sarraf D (2016). Retinal Capillary Density and Foveal Avascular Zone Area Are Age-Dependent: Quantitative Analysis Using Optical Coherence Tomography Angiography. Invest Ophthalmol Vis Sci.

[28] Magrath, G. N. *et al*. Variability in Foveal Avascular Zone and Capillary Density Using Optical Coherence Tomography Angiography Machines in Healthy Eyes. *Retina*, doi:10.1097/IAE.0000000000001458 (2016). 10.1097/IAE.0000000000001458 27997512

[29] Kuehlewein L (2015). Noninvasive Visualization and Analysis of the Human Parafoveal Capillary Network Using Swept Source OCT Optical Microangiography. Invest Ophthalmol Vis Sci.

[30] Staurenghi G (2015). Optical coherence tomography angiography of the retinal microvasculature using the Zeiss AngioPlex. European Ophthalmic Review.

